# Diagnosis and Early Management of Robin Sequence

**DOI:** 10.3390/children11091094

**Published:** 2024-09-06

**Authors:** Alexander J. Rickart, Oishi Sikdar, Allan Jenkinson, Anne Greenough

**Affiliations:** Department of Women and Children’s Health, Faculty of Life Sciences and Medicine, King’s College London, London SE5 9RS, UK

**Keywords:** prenatal diagnosis, Pierre Robin syndrome, airway obstruction

## Abstract

The results of a survey of twenty-four neonatal units in the United Kingdom and Ireland are presented. A structured ten-item questionnaire was used, and demonstrated the variation in how infants with RS are diagnosed and managed. Notably, the survey revealed that a minority of infants were diagnosed antenatally. There were significant discrepancies in diagnostic criteria used and 79% of the units referred the patients to tertiary services. A preference for minimally invasive approaches to managing upper airway obstruction, such as a trial of prone positioning before progressing to a nasopharyngeal airway, was reported by 96% of the centers. A narrative review was undertaken which discusses the current practices for diagnosis and early management of Robin sequence (RS). The challenges of antenatal diagnosis, strategies to enhance outcomes through early detection and controversies surrounding the management of neonatal upper airway obstruction associated with RS are included. The results of the survey and our comprehensive review of the literature emphasize that there remains uncertainty regarding the best approach to treating Robin sequence.

## 1. Introduction

Robin sequence (RS) is characterized by a clinical triad of micrognathia, glossoptosis, and upper airway obstruction (UAO). The incidence of RS ranges from 1 in 8000 to 1 in 20,000 live births, depending on the diagnostic criteria used [1,2,3,4,5,6]. First described in 1923, this congenital condition arises from the underdevelopment of the mandible, which leads to retropositioning of the tongue and subsequent airway obstruction [7]. The resulting difficulties with breathing and feeding are often complex to manage, necessitating multidisciplinary specialist care [1,8].

Despite nearly a century of recognition, the optimal approach to the management of RS remains equivocal. There are significant variations in clinical practice both nationally and internationally, compounded by a lack of consensus among clinicians and within clinical guidelines. This presents unique challenges for those involved in the perinatal care of these children, where critical decisions impacting both short- and long-term outcomes are made. In this context, providing clear and consistent information to caregivers is essential for supporting them in shared decision-making and to achieve better outcomes.

We conducted a survey of neonatal units in the United Kingdom and Ireland, underpinned by a narrative review to illustrate current clinical issues, instances of equipoise, and emerging trends in the diagnosis and management of Robin sequence.

## 2. Materials and Methods

### 2.1. Survey

A ten-item structured questionnaire (Table 1) which focussed on diagnosis, assessment, and management of Robin sequence was created. A consultant neonatologist, senior specialty registrar, or advanced neonatal nurse practitioner at each of the 211 neonatal units in the United Kingdom and Ireland were contacted, initially by email then by follow-up telephone call.

### 2.2. Narrative Review

The search strategy and selection criteria for the narrative review included a comprehensive review of MEDLINE, Pubmed and Web of Science with the key search terms of “Pierre-Robin”, “Glossoptosis”, “Upper Airway Obstruction” and “Micrognathia”. The initial search yielded 490 articles, and the abstracts were screened, prioritizing recent publications with comprehensive datasets, thorough analysis and relevance to current clinical practice. We cross-referenced both the International Paediatric Otorhinolaryngology Group and the European Reference Network Guidelines on Robin sequence. One hundred and thirty-three papers were selected by consensus of the authors.

## 3. Results

Twenty-four units responded to the survey. Of the respondents, 8% were from level one units, 54% from level two local neonatal units, and 38% from level three neonatal intensive care units. At 85% of these units, between one and five children with RS were born each year, with the remainder seeing the condition less frequently. The low response rate, compared to previous surveys conducted by our unit [9,10], may reflect a lack of exposure to such infants in many centers. but is also a limitation in capturing the breadth of current practice. Regardless, it is recommended that their care be centralised in specialist units, ideally following antenatal diagnosis. Table 1 provides a summary of the questionnaire and the corresponding responses.

## 4. Discussion

Our survey findings highlight the variability in the care provided to infants with RS across units in the United Kingdom and Ireland. Due to the limited exposure to RS, it is recommended that care be centralized in tertiary units, ideally following antenatal diagnosis. This approach ensures that children receive optimal care at the appropriate time, whether in the delivery room, during assessment, or if intervention is required. Drawing on our survey results, we outline the early care pathway for infants with RS and compare those practices with the existing literature.

## 5. Diagnostic Criteria and Classification

The current diagnostic criteria for RS include a combination of micrognathia, glossoptosis, and upper airway obstruction [1,4,11,12]. These anomalies develop sequentially in utero, where poor mandibular development leads to glossoptosis, subsequently obstructing the upper airway in the neonatal period. The retropositioning of the tongue can inhibit the fusion of the palatal shelves, resulting in a cleft palate, which is found in the majority (80–90%) of RS patients [12,13,14,15,16,17]. As a cleft palate is not always present, it is not part of the formal diagnostic criteria. Historically, there have been discrepancies in the observed criteria, which was reflected in our survey. All respondents identified micrognathia as an essential feature. There was, however, a lack of consensus on the inclusion of glossoptosis, upper airway obstruction, and cleft palate, with only two-thirds of respondents reporting the correct triad.

A key distinction to consider is that RS can be broadly classified as isolated or syndromic. In isolated cases, the core triad are present with or without a cleft palate, but with no other additional features or genetic abnormalities. Syndromic cases involve RS either with additional clinical features, genetic abnormalities or a combination of the two. Commonly recognized associations include Stickler, Treacher-Collins, and 22q.11.2 deletion syndromes [18,19,20]. It is estimated that nearly half of all cases are isolated, but this should be considered in the context of low patient numbers and the varying methods of genetic testing [1,14,15]. Antenatal genetic testing is more nuanced and will be discussed later, but those born with RS should be referred to a clinical geneticist. After appropriate counseling, tailored screening can be conducted, with whole genome sequencing being the gold standard. In the United Kingdom, this would currently form part of the Generation Study which builds on the foundations of the 100,000 Genomes Project [21]. Accurate genetic diagnosis aids in tailoring further investigations and treatments and facilitates discussions about risks in future pregnancies.

## 6. Antenatal Diagnosis

Fetal anomaly screening programs help to identify those who may benefit from specialist input or treatment before or after birth. Furthermore, they can aid in appropriately counselling parents and prompting discussions regarding prognosis and termination of pregnancy. It is possible to screen for RS antenatally, but currently this reflects less than a third of reported cases [22]. This was mirrored in our survey where 58% of respondents noted that a minority of cases had an antenatal diagnosis, representing an area with significant potential for improvement.

### 6.1. Ultrasound

Features that are highly suggestive of RS can be identified on a 20-week fetal anomaly scan, although diagnosis can only be confirmed postnatally. There have been a number of studies reporting that a more acute inferior facial angle, frontal nasal mental angle, or facial mental angle can be predictive of RS [23,24,25,26,27]. Similarly, a comparison of mandibular length with respect to gestational age can be used [28]. Sensitivities of these linear methods range from 84–95%, with specificities reported between 81–95%. Unfortunately, integration into routine clinical care is hindered by the rarity of the condition, the need for specialist knowledge, and manual post-processing of imaging data [29]. 

Recent advancements in imaging and artificial intelligence have shown the potential to automate extraction of fetal biometrics from ultrasound recordings [30,31]. Combining novel approaches to improve the processing of routine data holds great potential in the early antenatal detection of micrognathia, using a cost-effective and widely utilized imaging modality. 

### 6.2. Fetal MRI

As a benefit of research into antenatal imaging of the fetal brain and heart, there are increasing resources available to undertake fetal MRI [32,33]. In line with this, improved imaging protocols and development of services means that the indications for detailed cross-sectional imaging are increasing. Craniofacial anomalies, with micrognathia and RS included in this, are one of these indications. For those with suspected micrognathia on ultrasound, follow-up imaging at a specialist center with fetal MRI can improve the understanding of the severity of the condition. Particular advantages include the ability to assess the degree of glossoptosis and identify instances of isolated cleft palate, which can be challenging to detect on ultrasound [34,35,36,37,38]. This additional imaging allows for better risk stratification and counseling of parents.

### 6.3. Genetics

There is growing interest in conducting more detailed genetic testing during the antenatal period, particularly for pregnancies where routine screening protocols have highlighted a possible anomaly. In addition to the aforementioned ultrasound and MRI features, the presence of polyhydramnios may help distinguish between syndromic and isolated cases and influence the decision to proceed with further genetic testing [39,40]. Chromosomal microarray analysis has the ability to detect pathogenic copy number variations and is a useful addition to karyotyping, increasing the frequency of detection of abnormalities [41,42,43]. While whole genome sequencing (WGS) has been explored in the antenatal setting, whole exome sequencing (WES) may be a more practical alternative [44]. WES and targeted genetic panels provide a more efficient process, offering lower costs and faster results [44,45,46,47]. This is particularly relevant in the antenatal setting, where WES identifies diagnostic genetic variants in only 8.5% of cases where structural anomalies were detected on fetal ultrasound [42]. Given the complex ethical implications and resource intensive nature of genome-wide sequencing antenatally, this might be best reserved for cases with multiple anomalies and following appropriate counseling and genetic review [1,22,42].

### 6.4. Counseling

Children with RS often experience breathing and feeding difficulties during the neonatal period and will likely require ongoing multidisciplinary care. Offering parents detailed information, along with options for further imaging and comprehensive genetic testing represents the current best standard of care [1]. These evaluations should be conducted promptly, enabling parents to make informed decisions about continuing the pregnancy and help them adequately prepare for the potentially challenging postpartum period. The key to this lies in improving early detection on routine ultrasound which facilitates early referral to tertiary centers for expert-led care. 

Analysis of parental experiences of counseling in RS highlighted that antenatal diagnosis aided understanding of the condition and adjustment to the potential difficulties both parent and child might encounter. Conversely, some parents expressed that early diagnosis increased their anxiety during this period and found neonatal genetic counseling difficult to take in or overwhelming, especially if their child remained in hospital for the first weeks of life [22]. As there are suggestions that an antenatal diagnosis of RS correlates with a more severe phenotype, the need for comprehensive counseling becomes compelling [23,24,25]. 

### 6.5. Delivery

Given the potential for individuals with RS to require immediate management of a difficult airway, it is prudent that such cases be delivered in a specialist tertiary center [1,8,48,49]. Interestingly, our survey revealed that, due to the low rate of antenatal diagnosis, several infants with RS were born in centers lacking sufficient expertise for emergent neonatal airway issues. While 92% of units had guidelines for managing a difficult neonatal airway, only 13% had a dedicated pediatric difficult airway team. Additionally, only half of the units had specialist pediatric anaesthetic cover and a third had onsite ear, nose, and throat surgical cover. Those findings underscore the importance of antenatal diagnosis. In cases where concerning features are identified, it would be appropriate to discuss daytime delivery via a planned caesarean section with the relevant specialties in attendance. 

## 7. Assessment

The primary issue guiding treatment in RS is upper airway obstruction (UAO). After stabilization, a methodical approach should be taken to assessment. Early clinical evaluations might not always reveal signs of UAO, but this does not rule out the possibility of the child encountering problems later [50]. Even when initial assessments using pulse oximetry and capillary blood gases appear reassuring, repeated evaluations over the first weeks and months of life are essential. Breathing difficulties often fluctuate and may only become evident during sleep, feeding or periods of distress. Clinical observations alone, however, are insufficient; for instance, only 55% of infants with RS and obstructive sleep apnea (OSA) snore [51]. As a result, polysomnography is recommended in all infants where RS is suspected [1,8,52]. 

Our survey revealed that the majority of centers use a combined clinical assessment along with pulse oximetry and capillary blood gases to evaluate infants with RS. Seventy-nine percent of those centers, however, referred cases of suspected RS to tertiary services. As a result, only 38% of the units employed flexible nasoendoscopy to assess UAO and 25% offered polysomnography.

### 7.1. Polysomnography

Although polysomnography (PSG) is widely used to aid in diagnosing OSA, interpretation in the neonatal and infant population can be challenging [53]. Changes in oxygen saturation and the apnea-hypopnea index (AHI) are most commonly used to quantify the severity of OSA. Normative values, however, are taken from older children, with commonly referenced cut-offs categorizing the severity of OSA as mild (AHI from 1 to less than 5), moderate (AHI from 5 to less than 10), and severe (AHI greater than 10) [52]. Neonate specific ranges for PSG and nocturnal oximetry are insightful but based on studies with small sample sizes. The upper reference value for mixed obstructive AHI ranges from 5.8 episodes per hour at the age of one month old to 3.6 episodes per hour for a one-year-old, making the aforementioned ranges a point of contention [53,54,55,56,57]. 

A recent systematic review assessing the severity of UAO in RS highlighted the lack of consistency with which UAO was assessed and discrepancies between thresholds set for intervention [58]. Given the above, the interpretation of PSG results must be considered in a holistic context, rather than numerical data with predefined thresholds intended to directly influence clinical decision-making. In the future, a key research priority should be to obtain a clearer understanding of normal breathing patterns during sleep in the first year of life. We recommend the implementation of standardized reporting in future research. This approach should be specifically tailored to the RS population, involving extended monitoring of both diurnal and nocturnal sleep patterns and critically differentiating between obstructive and central apneas.

### 7.2. Flexible Nasoendoscopy

Additional methods for assessing the degree of upper airway obstruction (UAO) include flexible nasoendoscopy (FNE) and drug-induced sleep endoscopy (DISE) [59,60,61,62]. These techniques should be integrated into clinical care to evaluate both the extent and the location of UAO, particularly in complex or syndromic RS cases where multilevel obstruction may be present [59]. 

### 7.3. Feeding and Growth

The same anatomical factors that contribute to UAO can disrupt the suck-swallow-breathe reflex, leading to subsequent feeding difficulties. As a result, gastroesophageal reflux, altered esophageal tone, and an increased risk of aspiration are frequently observed in RS [63,64,65,66,67]. Given these challenges, early involvement of speech and language therapists is beneficial for both the parents and child. Early assistance with breast feeding or the use of specialist feeding bottles is particularly valuable when a cleft palate is present [68]. Additionally, it is important to observe feeding closely, as dysfunctional feeding has the potential to exacerbate respiratory compromise.

Infants with RS have a limited reserve and greater metabolic demands due to the increased work of breathing. Close monitoring of growth and development can help identify those who may need additional support. Concerns regarding failure to thrive would prompt further investigation, with select cases benefitting from electromyography, esophageal pH monitoring, or FNE [65,66,69].

Enteral feeding is reported to be required in 42–82% of cases, with nasogastric tube placement being required for a median duration between 14–30 weeks [14,70,71,72]. Interestingly, there does not appear to be a direct correlation between phenotypic severity and the need for long-term enteral feeding [71,73]. The incidence of enteral feeding is similar in both isolated and syndromic RS groups. A small proportion of patients (between 7 and 15%) require longer-term feeding through a gastrostomy [71]. Ultimately, optimizing the airway, whether through minimally invasive techniques or surgical management, will secondarily address the feeding issues [72]. 

### 7.4. Decision-Making

Despite various proposed treatment algorithms, classifications, and consensus statements, there is still a lack of agreement on best practices. These tools, although beneficial for research, do not sufficiently address the nuances of clinical practice, especially given the strength of evidence behind them [1,8,74,75,76,77,78,79,80,81,82]. Instead, given that RS encompasses such a wide-ranging phenotype, it would be pragmatic for all children with RS to undergo multimodal assessment in high-volume tertiary centers. Following initial stabilization and assessment, the multidisciplinary team should discuss and adapt the treatment strategies, focusing on the specific needs of the patients and their parents [83].

## 8. Management

### 8.1. Minimally Invasive Management

Initial trials of lateral or prone positioning may be beneficial for managing patients with RS, as this method was reported to be sufficient in the majority of cases. The criteria for determining the success of treatment vary, but they generally focus on one or more of the following outcomes: resolution or improvement of airway obstruction, adequate nutrition and growth and the avoidance of the need for further treatment. Although most studies are limited to retrospective cohorts, it is reported that between 47 and 100% of patients can be adequately managed with positioning alone [72,84,85,86,87,88,89,90]. Recently, its efficacy has been called into question and it remains controversial [89,91,92]. While it appears a pragmatic short-term measure, patients who require ongoing positioning to alleviate airway obstruction should be considered for further intervention due to the increased incidence of sudden infant death syndrome (SIDS) associated with prone positioning [93]. Therefore, positioning can be attempted in the first instance, but the associated risks must be clearly communicated to parents and monitoring may be of benefit [94].

For patients where positioning is insufficient, the insertion of an NPA is a simple, well-tolerated and effective option to bypass UAO. An NPA can serve as a temporary measure or a second-line treatment following a failed attempt at positioning. If successful in alleviating airway obstruction, this can facilitate discharge into the community, provided there is appropriate education of care-givers and close follow-up [95]. Studies have shown that NPA is effective in overcoming both moderate and severe UAO, with reported success rates between 60 and 100% [72,84,85,87,90,95]. An alternative to NPA is the pre-epiglottic baton plate (PEBP). This orthodontic device consists of an acrylic plate that lies along the palate with an oropharyngeal extension designed to bring the tongue base anteriorly and open the airway [96]. Requiring significant expertise, there are only a few units utilizing this method, with the majority of the research from a single center in Tübingen, Germany. There are, however, promising and well documented results that show relief of UAO on PSG and improved feeding [96,97,98,99,100,101]. Both PEBP and NPA address obstruction at the level of the tongue base, but there is a dentofacial orthopedic component to the PEBP that is of particular interest [102,103,104]. As such, a direct comparison between these two minimally invasive methods would be of significant value, especially with regards to mandibular growth [105]. 

Both high-flow nasal cannula and continuous positive airway pressure have the ability to manage the UAO encountered in RS. These therapies can be safely administered to infants in community settings, depending on local expertise, or used as bridging measures when necessary [96,106]. Combining an NPA with CPAP is also an alternative approach to airway management [107]. Limitations of CPAP, however, include the difficulty in maintaining an adequate seal alongside a nasogastric tube and the risk of pressure injuries.

Our survey revealed a trend towards conservative or minimally invasive management. Specifically, 96% of units were trialing prone positioning, with the same percentage progressing to a NPA if necessary. Additionally, 42% and 46% of units would consider using high-flow nasal cannula or continuous positive airway pressure, respectively. All respondents agreed that only a small minority of patients require endotracheal intubation. While this procedure can be life-saving, it is not a definitive solution. For patients who do not respond to treatment or who urgently need a secure airway, surgical management should be considered.

### 8.2. Surgical Management

The three primary options for surgical management are tracheostomy, tongue-lip adhesion (TLA), and mandibular distraction osteogenesis (MDO). Less invasive treatments are more common in European centers, whereas North American centers tend to show a stronger preference towards MDO [4,78,108,109]. Treatment approaches are largely influenced by the philosophy and experience of the operating center. In select populations, MDO can be employed to avoid tracheostomy. Alternatively, tracheostomy can serve as a temporary measure while the infant grows, with MDO being utilized only in cases where decannulation is unsuccessful. The third option, TLA, is less frequently used and involves an invasive procedure to draw the base of the tongue forward by passing a deep suture through the tongue and securing it to the lower lip. Cases in which this would be effective, however, significantly overlap with those that could be managed by NPA or PEBP [110,111,112,113,114,115,116]. 

MDO employs the principle of distraction osteogenesis, first introduced in orthopedic surgery by Ilizarov and later adapted for craniofacial surgery by McCarthy [117,118,119]. This technique enhances the anteroposterior projection of the mandible, effectively addressing the underlying pathology in RS. A number of predominantly retrospective studies have reported positive outcomes, including relief of airway obstruction, facilitation of decannulation (80–92% in both isolated and syndromic RS) and improved nutritional intake [120,121,122,123,124,125,126,127,128,129,130]. There remains, however, a small subgroup of RS patients for whom even repeated MDO is insufficient to facilitate decannulation. It is worth noting that MDO has a substantial burden of care and is not without complications, including potential long-term effects on dentition and mandibular growth [131,132,133]. 

## 9. Conclusions

There remains uncertainty regarding the best approach to treat Robin sequence. The establishment of an international observational registry to document the epidemiology, treatment, and outcomes of RS patients would be helpful [105], with the aim of advancing the understanding of RS and improving patient care. Future research should prioritize the following key areas:

- ***Integrating artificial intelligence into antenatal screening:*** this technology has the potential to improve the rate of antenatal diagnosis.
- ***Expanding the understanding of the Robin sequence genotype:*** enrolling patients in genomics studies focusing on rare diseases will deepen our understanding of RS.
- ***Advancing the knowledge of neonatal sleep physiology:*** establishing age-appropriate normal values and standards will facilitate fair comparison and appraisal of interventions.
- ***Comparing minimally invasive and surgical techniques:*** further research is needed to determine the optimal thresholds for intervention.

## Figures and Tables

**Table 1:** Questionnaire and Summary of Responses.

**What is the highest level of care provided at your unit?**	**Level 1**			**Level 2**				**Level 3**		
	8.3%			54.2%				37.5%		
**How many infants with RS are born in your hospital** **each year?**	**0**			**1–5**				**5–10**		
	15.4%			84.6%				0%		
**Of the patients presenting to your unit with RS, approximately how many have had an antenatal diagnosis made on ultrasound?**	**Majority**					**Minority**				
	41.7%					58.3%				
**Which of the following criteria does your unit use to** **make a diagnosis of RS? Please select all that apply.**	**Micrognathia**		**UAO**			**Glossoptosis**			**Cleft Palate**	
	100%		66.7%			66.7%			66.7%	
**Does your unit have a guideline for the management of** **difficult neonatal airways?**	**Yes**					**No**				
	91.7%					8.3%				
**If your unit has experience in managing difficult neonatal airways, which of the following teams are available at birth? Please select all that apply.**	**ENT**		**ENT cover** **off site**			**Pediatric** **Anaesthetics**			**Airway Team** **For Neonates**	
	33.3%		62.5%			50%			12.5%	
**How does your unit assess the airway in patients with RS?** **Please select all that apply.**	**Clinically**	**Oximetry** **& CBG**			**FNE**		**PSG**			**Referral**
	95.8%	83.3%			37.5%		25%			79.2%
**For patients with airway obstruction that is not immediately life threatening, does your unit trial prone positioning?**	**Yes**					**No**				
	95.8%					4.2%				
**If you do not use prone positioning or it does not relieve the airway obstruction, what is your next line of treatment? Please select all that apply.**	**NPA**			**HFNC**				**CPAP**		
	95.8%			41.7%				45.8%		
**How many infants with RS require intubation to acutely** **manage their airway?**	**Majority**					**Minority**				
	0%					100%				

UAO—upper airway obstruction; CBG—capillary blood gas; FNE—flexible nasoendoscopy; PSG—polysomnography; NPA—nasopharyngeal airway; HFNC—high flow nasal cannula; CPAP—continuous positive airway pressure.
